# Gazepath: An eye-tracking analysis tool that accounts for individual differences and data quality

**DOI:** 10.3758/s13428-017-0909-3

**Published:** 2017-06-07

**Authors:** Daan R. van Renswoude, Maartje E. J. Raijmakers, Arnout Koornneef, Scott P. Johnson, Sabine Hunnius, Ingmar Visser

**Affiliations:** 1 Department of Psychology, University of Amsterdam, Amsterdam, The Netherlands; 2 Department of Education and Child Studies, Leiden University, Leiden, The Netherlands; 3 Department of Psychology, University of California, Los Angeles, CA USA; 4 Department of Psychology, Radboud University, Nijmegen, The Netherlands; 5 Research Priority Area Yield, University of Amsterdam, Amsterdam, The Netherlands; 6 Amsterdam Brain and Cognition, University of Amsterdam, Amsterdam, The Netherlands

**Keywords:** Infant eye movements, Eye-tracking methodology, Fixation duration, Attention, Event detection

## Abstract

Eye-trackers are a popular tool for studying cognitive, emotional, and attentional processes in different populations (e.g., clinical and typically developing) and participants of all ages, ranging from infants to the elderly. This broad range of processes and populations implies that there are many inter- and intra-individual differences that need to be taken into account when analyzing eye-tracking data. Standard parsing algorithms supplied by the eye-tracker manufacturers are typically optimized for adults and do not account for these individual differences. This paper presents gazepath, an easy-to-use R-package that comes with a graphical user interface (GUI) implemented in Shiny (RStudio Inc 2015). The gazepath R-package combines solutions from the adult and infant literature to provide an eye-tracking parsing method that accounts for individual differences and differences in data quality. We illustrate the usefulness of gazepath with three examples of different data sets. The first example shows how gazepath performs on free-viewing data of infants and adults, compared to standard EyeLink parsing. We show that gazepath controls for spurious correlations between fixation durations and data quality in infant data. The second example shows that gazepath performs well in high-quality reading data of adults. The third and last example shows that gazepath can also be used on noisy infant data collected with a Tobii eye-tracker and low (60 Hz) sampling rate.

**Electronic supplementary material:**

The online version of this article (doi:10.3758/s13428-017-0909-3) contains supplementary material, which is available to authorized users.

## Introduction

Eye-tracking has become a popular tool in many psychological disciplines. For instance, eye-tracking is used to study reading abilities (Rayner, Castelhano, & Yang, 2009) and real-world scene perception (Henderson 2003) in different types of populations and age groups. For example, eye-trackers enable researchers to quantify differences between clinical populations and healthy controls in disorders such as schizophrenia, attention-deficit hyperactivity disorder (ADHD) and Williams syndrome (e.g., Riby & Hancock, 2008; Karatekin & Asarnow, 1999). Even in infants, looking measures have been suggested to predict infants at risk of developing autism (Wass et al. 2015). In reading research, eye-tracking can provide insights into reading behavior differences between children with and without dyslexia (e.g., Hutzler & Wimmer, 2004), or between children, adults, and the elderly (Paterson, McGowan, & Jordan, 2013; Reichle et al., 2013; Rayner, Reichle, Stroud, Williams, & Pollatsek, 2006; Rayner et al., 2009).

The fact that eye-tracking can be used in such a broad range of populations is one of its main advantages (Karatekin 2007). However, this also implies that there are most likely individual differences that should be taken into account, especially when comparing different populations. This paper presents gazepath: an R-package developed to detect fixations in eye-tracking data while accounting for individual differences.

Fixations and saccades are the main elements of gaze patterns. During fixations, visual processing takes place and encoding information in memory is possible, whereas saccades are the rapid eye movements during which visual sensitivity is suppressed (Matin 1974). In order to analyze gaze patterns, eye-tracking data must be parsed into fixations and saccades. This is commonly accomplished by using dispersion, velocity, and/or acceleration-based algorithms supplied by the eye-tracker manufacturer. For example, EyeLink (SR Research Ltd., Ontario, Canada) uses a velocity threshold of 35 deg/s and an acceleration threshold of 8000 deg/s ^2^ as default values, although these thresholds can be altered manually. When both speed and acceleration of the eye exceed these thresholds, it is assumed that a saccade took place. Dispersion thresholds, on the other hand, assume that a saccade takes place when a distance threshold is crossed. For instance, the Tobii Clearview 2.7 Tobii Eye Tracker User Manual (2006) defines the end of a fixations when the eye has moved .9 ^∘^ of visual angle, although this threshold can also be set to different values.

In our eye-tracking studies with infants (Van Renswoude, Johnson, Raijmakers, & Visser, 2016), we noticed that these standard algorithms with fixed thresholds were often unable to correctly identify fixations and saccades. This is a well-known problem in infant eye-tracking research (e.g., Wass, Forssman, & Leppänen, 2014; Hessels, Andersson, Hooge, Nyström, & Kemner, 2015; Gredebäck, Johnson, & von Hofsten, 2009), as well as in adult eye-tracking research (e.g., Shic, Scassellati, & Chawarska, 2008; Nyström & Holmqvist, 2010). The aim of this work is to combine solutions from the fields of adult and infant eye-tracking and develop a tool that can be used to parse eye-tracking data of different populations and data quality into fixations.

### Individual differences

Standard velocity and dispersion thresholds provided by eye-tracker manufacturers are not always optimal. Sometimes small saccades are missed because the threshold was not crossed, and it also happens that a speed and/or dispersion threshold is crossed, while no actual saccade took place. Optimizing the detection of fixations requires the use of different thresholds for different participants. Even in different blocks or trials, stimuli, tasks, or the mood of the participant can elicit different eye movements that are best classified by different thresholds. Standard algorithms supplied by eye-tracker manufacturers assume one threshold for everyone at every time during the experiment.

Setting individual thresholds can possibly improve fixation detection, although there are some drawbacks. For instance, in a study it could become difficult to tell whether observed individual differences on the task reflect real underlying differences, or an artifact of the different threshold choices. Study results can depend on these threshold choices. Shic et al. (2008) showed that using a different threshold, but the same within groups, can result in the (dis)appearance of an effect between these groups. The use of individual thresholds also complicates the replication and comparison of these studies (Nyström & Holmqvist, 2010). Therefore, statistical criteria are needed to define threshold values.

The literature offers several data-driven algorithms for defining thresholds (e.g., Blignaut, 2009; Shic et al., 2008; Nyström & Holmqvist, 2010). In a recent paper, Andersson, Larsson, Holmqvist, Stridh, and Nyström (2016) compared ten (mostly data-driven) algorithms with classification by humans. The aim of their study was to find the best performing algorithm, but they found large differences in performance, making it difficult to determine the best. Applied to static stimuli, the adaptive velocity-based algorithm of Nyström and Holmqvist (2010) produced similar fixation durations as trained human coders. On a sample-to-sample basis, however, other algorithms performed well. For instance, algorithms that use hidden Markov models (Komogortsev, Gobert, Jayarathna, Koh, & Gowda, 2010), a binocular-individual threshold (van der Lans, Wedel, & Pieters, 2011) or a simple velocity threshold had also a close match to the human coders. An algorithm that Andersson et al. (2016) did not take into account is the algorithm developed by Mould, Foster, Amano, and Oakley (2012). This velocity-based algorithm is completely data-driven, meaning there is no need for initial starting values as in most data-driven algorithms. The Mould et al. (2012) algorithm is able to adapt itself to the quality of the data by increasing velocity thresholds in low-quality data and lowering velocity thresholds in high-quality data. This algorithm makes it possible to apply the same method to the data of all participants, yet allowing for individual threshold estimation. This algorithm is developed for use in adult studies and not yet tested with infant data. Moreover, additional processing of the data is needed to deal with specific data-quality issues often observed in infants. As noise is a major issue in infant eye-tracking, we used the Mould et al. (2012) algorithm as a starting point for gazepath because this algorithm is explicitly designed to adjust thresholds to noise in the data without specifying an initial starting threshold.

### Data quality

A typical case of infant eye-tracking data is much noisier than adult eye-tracking data. Sampling point fluctuations are larger in infants than adults and there are much more missing sampling points. This is caused by multiple factors, for example, infants tend to make more head movements than adults, causing instances of missing data as the eye-tracker needs to re-identify the position of the head (Hessels et al. 2015). Head movements may also make it difficult for the eye-tracker to identify the eyes; for instance, the nostril may be mistaken for the pupil, resulting in a signal moving between the eye and the nostril. Furthermore, infants’ eyes can be watery, resulting in flicker in the data where the signal rapidly switches between on and off (Wass et al. 2014).

**Fig. 1:**
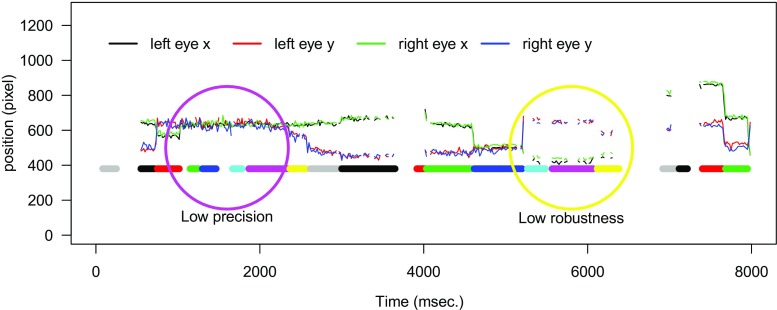
Example of low robustness and low precision in eye-tracking data collected with a Tobii 60 Hz eye-tracker. The *colored horizontal line* at y = 380, represents the fixations classified by the Tobii. When the color switches, a new fixation is identified, it can be seen that low data quality leads to identification of many short fixations. Also note the puzzling instances around 100 ms and 7000 ms, where Tobii detects fixations without any gaze data

Figure 1 shows 8 s of raw eye-tracking data measured with a Tobii eye-tracker (Tobii 1750, Tobii Technology, Stockholm, Sweden). Time is plotted on the x-axis and the x- and y-positions of the left and right eyes are plotted on the y-axis. Data quality is characterized by precision and robustness (Wass, Smith, & Johnson, 2013). Precision refers to the sampling point fluctuations. In Fig. 1 the signal in the purple circle shows large fluctuations, thus low precision. Robustness refers to sequences of missing data. When there is a constant signal, robustness is high, but when the signal flickers on and off, such as in the yellow circle in Fig. 1, robustness is low. The horizontal colored sequences below the left and right eye signals are the fixations that are classified by the standard Tobii event-detection algorithm. Each color change indicates a new fixation. In the purple circle, where precision is low, four fixations are classified; however by looking at the data, it seems more likely that one long fixation took place. Because of the low precision, the dispersion threshold of the Tobii algorithm is crossed several times and new fixations are classified. This shows how data quality can influence dependent variables such as fixation durations. In line with this example, Wass et al. (2014) found that data quality correlates with key dependent variables, such as fixation durations. Lower data quality goes hand in hand with shorter fixation durations. Furthermore, data quality is also affected by other variables, such as age. Older infants have better data quality than younger infants (Wass et al. 2014). This makes it hard to assess, for instance, the relationship between fixation duration and age, as it is also influenced by data quality (see Wass and colleagues, 2013, 2014 and Hessels and colleagues, 2015 for a more detailed discussion on data quality in infant eye-tracking.)

The relationship between data quality and dependent variables has been identified as a problem in infant eye-tracking studies, and several solutions have been offered. Wass et al. (2013), for example, developed a parsing algorithm that performs post hoc checks on the data. Fixations are only kept if they have incoming and outgoing saccades. This is done to make sure fixation durations are not affected by missing data instances. These algorithms were used as the basis of GraFIX, a semiautomatic approach for parsing eye-tracking data (de Urabain, Johnson, & Smith, 2015). A major advantage of GraFIX over most other algorithms is that GraFIX comes with a graphical user interface (GUI). This makes GraFIX also usable for researchers who lack MATLAB skills. A downside, however, is that GraFIX needs considerable user input. Fixations are initially parsed automatically and can then be manually adjusted. Despite these possible solutions, infant eye-tracking studies reporting data quality and/or taking measures to overcome the issues described here remain scarce.

### Current study

To summarize, standard eye-tracker manufacturer classification methods provide no satisfactory solution to reliably parse eye-tracking data of different populations, because they do not allow individual threshold estimation. The algorithms that use individual thresholds are not yet suited to analyze infant eye-tracking data and the algorithms developed by Wass et al. (2013) and de Urabain et al. (2015) to analyze infant data do not allow individual threshold estimation. Furthermore, most of these approaches (except GraFIX) are implemented in MATLAB, which is expensive and requires advanced programming skills to use. In this paper, we attempt to combine the best of both worlds into a new eye-tracking parsing tool called gazepath. Gazepath is an easy-to-use open-source software tool, implemented in R (R Core Team 2014). It comes with a GUI implemented in the R-package shiny (RStudio Inc 2015). Gazepath is capable of dealing with low-quality eye-tracking data in terms of robustness and precision, but is also well suited for high-quality data. We show this by examining correlations between data quality and outcome measures and assessing the distribution of fixation durations when the gazepath method is used, compared to the standard classification methods. The functionality of gazepath will be illustrated on different data sets; first, we show how gazepath performs compared to the standard EyeLink classification on a free-viewing data set of infants and adults. Second, we compare gazepath performance with EyeLink performance on an adult reading data set. Third, we illustrate how gazepath performs on low sampled (60 Hz) infant experimental data collected with a Tobii. These data sets are chosen to reflect the data extremes obtained with eye-trackers. On the one end of the spectrum, there is infant free-viewing, which can be highly variable without any predictable patterns to expect. On the other end, there is adult reading, a highly automatic process with a very predictable pattern.

## Gazepath method

The algorithm of Mould et al. (2012) is taken as basis for the gazepath package. This algorithm is able to account for individual differences by estimating a velocity threshold for every individual and every trial in a data-driven manner, thereby providing a perfect starting point to develop an algorithm that can be used for different populations. The algorithm also has some limitations, one of which concerns the estimation of the duration threshold. Although the algorithm is capable of doing this in a data-driven manner based on initial fixation durations, the duration threshold is too unreliable. We estimated the duration thresholds, leaving out one data point for every estimation. What we observed were threshold differences up to 50 ms. These are very large differences that cannot be justified with only a single data point difference. Another limitation is the ability to deal with low robustness in the data. Consequently, instances of missing data signal the end of a fixation, even if data is only missing for a few milliseconds. In order to overcome these limitations, we combined the Mould et al. (2012) algorithm with the methods described by Wass et al. (2013) into the R-package gazepath.

### Gazepath pre-processing

**Fig. 2:**
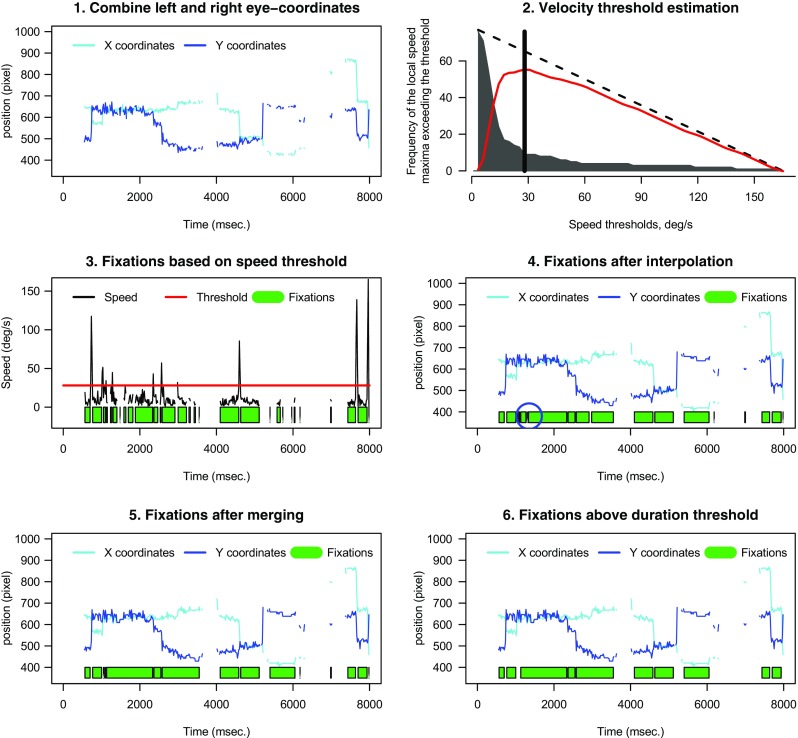
Example of all steps of the gazepath method. First, gaze coordinates are combined when two eyes where tracked. Second, the speed threshold is derived using the Mould et al. (2012) algorithm. Data sequences that fall below the threshold, are marked as initial fixations (see panel 3). Panel 3 also clearly shows the bad performance of initial parsing, as can be seen from the many short fixations that occur as a result of data quality. The fourth step is to interpolate sequences of missing data. Panel 4 shows this improves the classification a lot, but there are still instances (*blue circle*), where fixations should be combined. This is done in the fifth step, by combining successive fixations that overlap in space. The sixth and final step involves the selection of fixations that pass the duration threshold, which is often set to 100 ms

The gazepath method uses a six-step procedure to preprocess the data from raw samples into fixations and saccades. These six steps are described below and visualized in Fig. 2. First, raw data of the left and right eye are combined when two eyes were tracked. This is done by calculating the mean of the x- and y-coordinates. Missing data points from one eye are interpolated with data points of the other eye when possible. This is done to maximize the available data.

Second, the velocity threshold is estimated using exactly the same method as the Mould et al. (2012) algorithm to account for individual and trial-by-trial differences in precision. The velocity of the eye is calculated as the Euclidean distance between preceding and succeeding points divided by the time elapsed between them. Then, sampling points with velocities higher than the preceding and succeeding sampling point are classified as local maxima. The second panel of Fig. 2 shows the distribution of local speed maxima exceeding the threshold (gray histogram), compared to a uniform null distribution (Tibshirani, Walther, & Hastie, 2001) of local maxima exceeding the threshold (dotted line). The difference between these two distributions is given by the gap statistic (red line). This gap statistic is smoothed with a locally weighted quadratic regression (loess, Cleveland, 1979; Fan & Gijbels, 1996) with increasing bandwidths until the gap statistic reaches one maximum. This maximum is the velocity threshold.

Third, to account for low robustness, missing data sequences shorter than a given threshold (default = 250 ms) are interpolated. The default value is choosing so it is unlikely a saccade took place, as saccades take approximately 200 ms to program (Nyström & Holmqvist, 2010). This is only done when the velocity difference between the last measured sample before the missing data and the first measured sample after the missing data, does not exceed the velocity threshold. This is done to make sure no saccade took place during the loss of signal.

Fourth, data sequences of the interpolated data that are below the velocity threshold are marked as possible fixations and data sequences above the velocity threshold are marked as possible saccades. At this moment, it is still possible that there are fixations that are too short, because the velocity threshold was crossed without an actual saccade taking place.

Fifth, to correct these instances, a check is made for successive fixations overlapping in space. This is done by drawing a polygon around the fixations, and when two successive fixations have overlapping polygons, the fixations are merged into one fixation.

The sixth and final step is to remove short fixations. This is done by setting the duration threshold, the default value for which is 100 ms. Although the Mould et al. (2012) algorithm offers a possibility to do this in a data-driven manner, this requires a lot of data. In practice, especially in infant studies, there are rarely enough data to reliably estimate the duration threshold. For the final classification, the effect of the duration threshold is also limited, since relatively few fixations fall in the interval of 50–150 ms. Given these considerations, we decided to set the duration threshold manually.

### Using gazepath

This section describes the procedure to use gazepath. Gazepath is implemented in R (R Core Team 2014) and therefore requires the installation of R before gazepath can be used. In R, gazepath can be installed by running the commands:

$$\texttt{install.packages(`gazepath', dependencies = TRUE)} $$

$$\texttt{library(`gazepath')} $$

Gazepath can be used from the R command line, but there is also a Shiny (RStudio Inc 2015) application that provides gazepath with a GUI, which can be opened in a web browser with the command:

$$\texttt{GUI()} $$

**Fig. 3:**
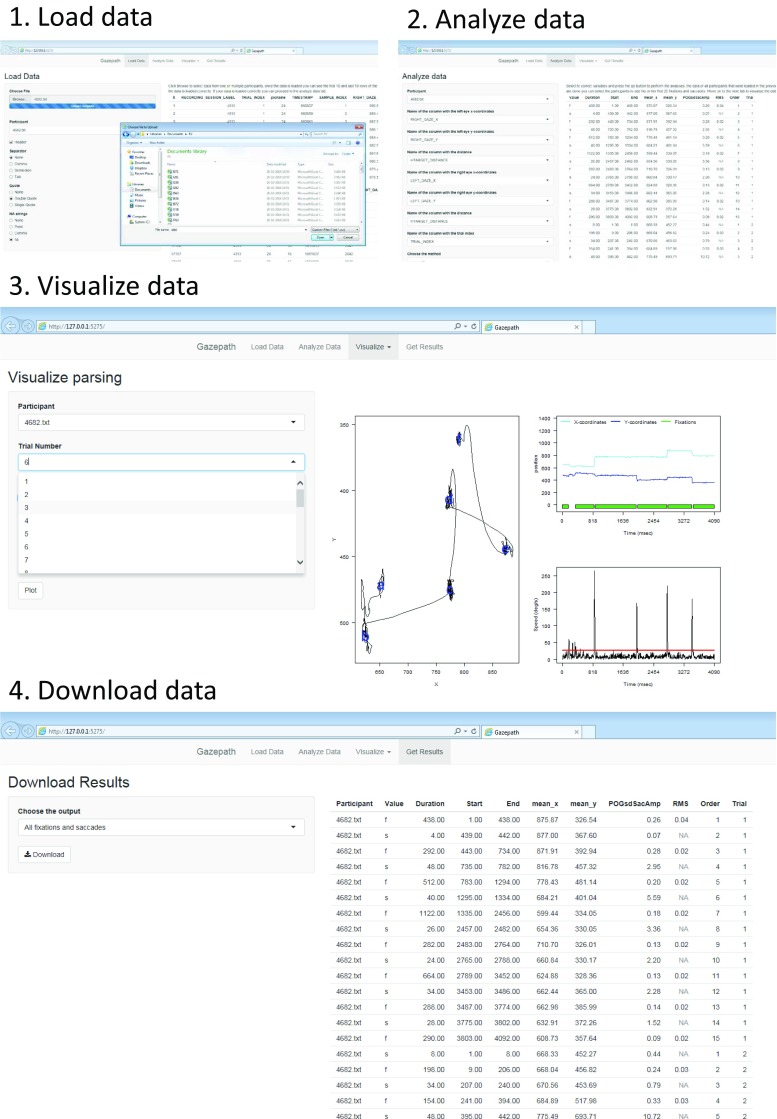
Illustration of the four-step procedure to parse fixations and saccades via the gazepath Shiny app

Here we use the Shiny app to illustrate the use of gazepath. First, the data are loaded; second, parsing takes place using the procedure described above; third, the data can be visualized; and fourth, the fixations can be downloaded (see Fig. 3).

After opening the application, the data must be loaded. Typically, eye-trackers generate text files with the raw data for every individual, and gazepath uses these files as input. As these text files can be formatted differently, there are several options to make sure the data are loaded correctly, such as different missing data strings and separation operators. On the right side of the screen, the top and bottom rows of the data file appear, and it is easy to check if the data are loaded correctly, i.e., if every point has its own cell in the data-frame. It is possible to load data of multiple participants, so the whole analysis can be conducted at once. However, loading multiple data sets requires all data sets to be formatted exactly the same way, i.e., having the same variable names, separation operators, etc.

Once the data are loaded, the next step is to provide gazepath with the information needed to run the analyses. From the uploaded data, gazepath needs at least the variable names of the x- and y-coordinates, distance to the screen and trial index. When two eyes are tracked, as is common with many trackers, the x- and y-coordinates and the distance to the screen of the other eye can also be specified. Furthermore, gazepath needs information about the screen dimensions in pixels and the stimulus dimensions in both pixels and mm (when stimuli presentation is not full screen, it is assumed that stimuli are presented in the middle). Finally, it is mandatory to specify the sampling rate and choose a parsing method. The best available methods are the gazepath and Mould methods, as described above. It is also possible to select the MouldDur method, which uses a fixed-duration threshold (default = 100 ms), the dispersion method, which is an implementation of the Tobii algorithm described in the Clearview 2.7 manual (Tobii Eye Tracker User Manual 2006), and the velocity method, which fixes the velocity threshold at 35 deg/s and the duration threshold at 100 ms. It is not recommended to use the last two methods. These methods are only implemented to ease comparison with simple parsing methods. Apart from the mandatory input, gazepath can keep other variables from the raw data, such as condition, age, stimuli, etc. These extra variables can only have a single value per trial, i.e., if different stimuli appear during one trial, the stimuli variable cannot be kept.

When all input parameters are set, the *go* button can be clicked to start the analysis. When there are multiple data sets loaded, this can take some time, and in the top right corner progress is displayed. It takes approximately 3 s to parse 1 min of 500-Hz data.^1^ After running the analyses, gazepath displays the top of the output file next to the input parameters. Now the data can be visualized. Fixations per participant per trial are displayed under *visualize parsing*, as seen in the middle of Fig. 3. The left screen plots the raw x- and y-coordinate overlaid with the order and position of fixations indicated by letters, the top right screen displays the raw x- and y-coordinates as the function of time and shows the fixations in green. The bottom right screen shows the speed in deg/s as a function of time with the velocity threshold in red. By clicking *visualize threshold* the velocity thresholds obtained for each individual on every trial are displayed. As estimation of the velocity threshold requires at least some data, some trials cannot be selected to inspect. This implies that there were not enough data to estimate a threshold in that trial. Finally, the fixations can also be visualized on the stimuli. Under the *visualize stimuli* tab, it is possible to upload the stimuli and plot fixations per participant per trial to inspect individual scanning patterns.

The final data can be downloaded as a .csv file, which can be used to further analyze the data. The data can be obtained in four forms, (1) all parsed fixations and saccades, (2) fixations only, (3) only complete fixations and saccades, i.e., fixations that have in- and outgoing saccades and saccades that are between two fixations, and (4) only complete fixations. The last two options can be selected to make sure all fixations and saccades are ‘true’ fixations and saccades; however, in noisy data this could result in much fewer data points. The fixations-only option can be useful, as most researchers are only interested in fixations. To close gazepath, simply close the browser and press esc in R to close the R process. The columns of the output data frame are ordered as follows:

Participant: the participant by the name of the data file.
Value: whether a fixation (f) or saccade (s) is classified.
Duration: the duration of the fixation or saccade in milliseconds.
Start and End: the start and end time in milliseconds of the fixations and saccades from the start of that trial.
mean_x and mean_y: the mean x- and y-coordinates in pixels of fixations and saccades (note that this measure is only meaningful for fixations).
sdPOGsacAMP: the standard deviation in point of gaze (for fixations) and the saccade amplitude in degrees of visual angle (for saccades).
RMS: the root mean square (RMS) within each fixation.
Order: the order of fixations and saccades within trials
Trial: the trial index.
*: When additional variables are kept from the original data, these variables appear after the last variable.

## Free-viewing data example

The performance of the gazepath method is examined in a free-viewing data set of infants and adults. This is an existing data set that is published elsewhere (Van Renswoude et al. 2016).

### Participants

Infant participants were recruited from Los Angeles County birth records. Adult participants were recruited through the University of California, Los Angeles subject pool and were given course credit for participating. Sixty-two infants (*M*_*age*_ = 9 months, range = 3–15) and 47 adults saw 28 real-world scenes for 4s each on a 17-inch computer monitor, which subtended an approximate 27^∘^× 34^∘^ visual angle. Eye movements were recorded with an EyeLink eye-tracker (SR Research Ltd., Ontario, Canada) that sampled at 500 Hz. Prior to data collection, a five-point calibration scheme was used to calibrate each participant’s point of gaze. The calibration procedure was repeated if necessary until the recorded point of gaze was within 1 ^∘^ of the center of the target.

### Descriptives

**Fig. 4:**
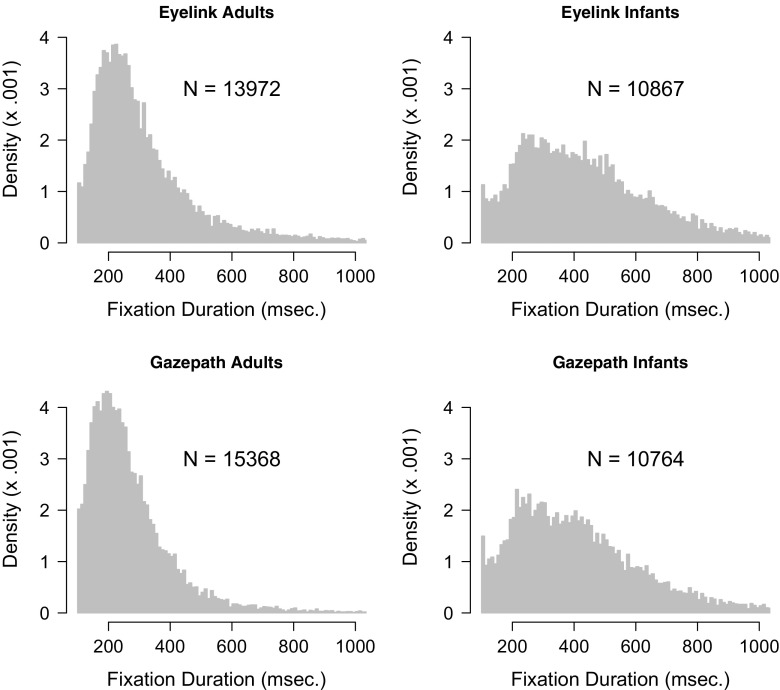
Distribution of fixation durations classified with the gazepath and EyeLink methods for free-viewing data of infants and adults. The distributions are plotted over a 100–1000-ms interval, whereas there are also some longer fixations classified

Fixations were detected by the gazepath method of the gazepath R-package and using the default settings of the EyeLink. Fixation durations typically show a right-skewed distribution, therefore the median fixation duration provides a more reliable measure than the mean (Helo, Pannasch, Sirri, & Raemae, 2014; Velichkovsky, Dornhoefer, Pannasch, & Unema, 2000). Figure 4 shows the distributions of the infant and adult free-viewing data parsed with the standard EyeLink and gazepath methods. Although the distributions look similar, there are some differences. The most striking difference is that fixations parsed by the standard EyeLink method are longer than the fixations parsed by gazepath. Another difference is the number of fixations. In adults, the gazepath method results in approximately 10% more fixations than the EyeLink method, whereas in infants the difference is only 1% and in the opposite direction.

**Fig. 5:**
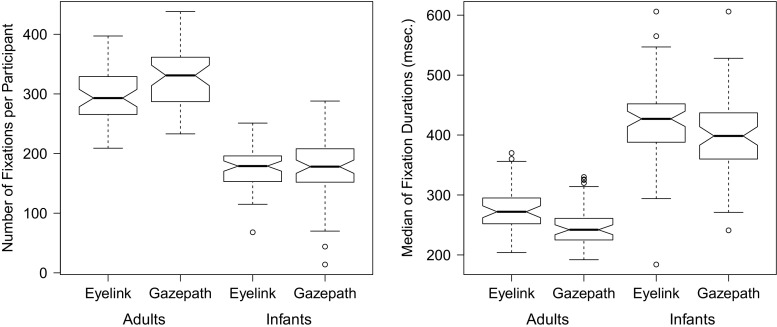
Boxplots of number of fixations (*left panel*) and median fixation durations (*right panel*) per participant, classified with the gazepath and EyeLink method for free-viewing data of infants and adults

In order to get a better understanding of these differences and to test the significance of these observations, the mean number of fixations and the median fixation durations were calculated for each participant. Figure 5 shows the boxplots of these means and medians for the infants and adults parsed by the gazepath and EyeLink methods. A factorial mixed ANOVA revealed an interaction effect between group (infant or adult) and method (gazepath or EyeLink) on the mean number of fixations, *F*(1,107) = 29.23,*p* < 0.001. For infants, there was no difference in the mean number of fixations classified by the EyeLink and gazepath method, whereas for adults the gazepath method classified more fixations than the EyeLink method. The median fixation duration differed between methods, *F*(1,107) = 108.75,*p* < 0.001. Fixations parsed using the gazepath method were shorter than fixations parsed with the EyeLink method. This difference was similar for infants and adults as there was no interaction effect between group and method for the median fixation durations, *F*(1,107) = 0.73,*p* = 0.396.

### Performance in adult data

For adults, these findings make sense, when fixations are shorter, more fixations can be made in the same time frame. This would imply that some fixations that are classified using the EyeLink method are split into two or more fixations using the gazepath method. This is likely, as gazepath sets the velocity threshold for every individual and every trial separately and lower thresholds would result in more fixations. To see if this is indeed what happened, we checked, for every fixation, for the possibility that the other method split that fixation.

**Fig. 6:**
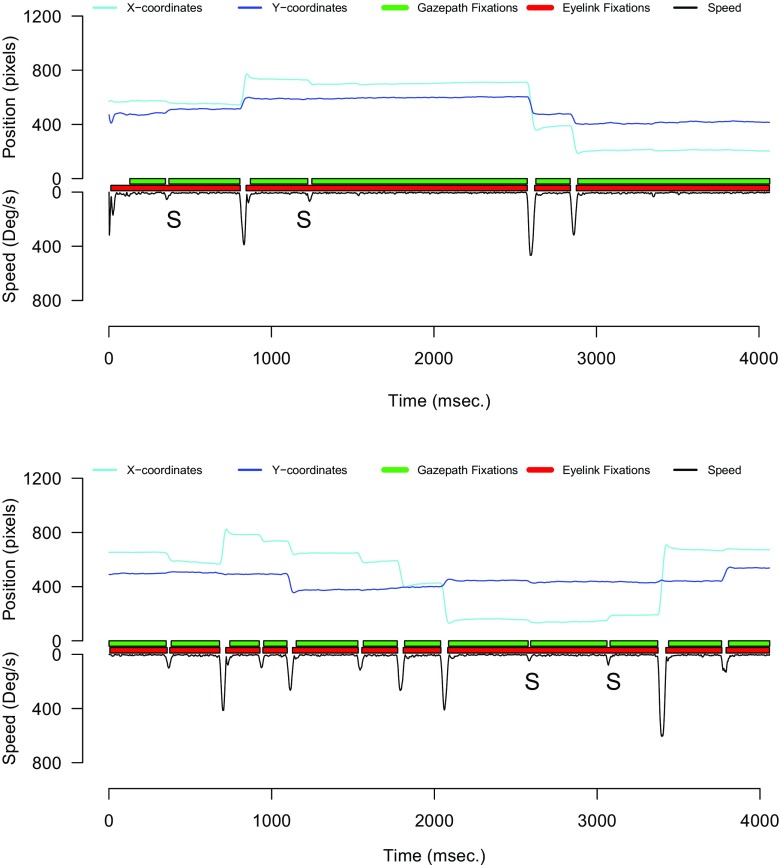
Example of gazepath and EyeLink classification of fixation. S denotes instances where a small saccade took place that is missed by EyeLink, but picked up by gazepath

Figure 6 provides a real data example wherein the letter S denotes saccades that led to splits. Here the gazepath fixations are identified as not being split, because every fixation also has one fixation classified by the EyeLink method. The first two EyeLink fixations are identified as being split because the gazepath method identified two fixations during the time frame of these fixations. Of the 10,764 gazepath fixations, only nine were split and only one fixation was not classified in the EyeLink method. Of the 10,867 EyeLink fixations, 1417 were split into 1738 extra fixations and 332 were not classified in the gazepath method. This explains the differences in the number of fixations between the two methods.

Answering the question of which method provides the best classification method is difficult, because it is impossible to establish a clear ground truth from the eye-tracking signal alone. Often classification by human experts is taken as the best available benchmark (e.g., Andersson et al., 2016). In order to get some insight into this question, we examined all trials in which there were one or more splits. Figure 6 shows two of these trials that are typical for what we observed. It can be seen that the gazepath method is more sensitive to small saccades (highlighted with S), which leads to more and shorter fixations being classified. Inspection of these trials also showed that most of the time the splits made in the gazepath method are easily observable by looking at the data, as is the case in these examples. However, we also observed trials where the splits were less prominent.

### Performance on infant data

For infants, the relationship between the number of fixations and the fixation duration is less clear than in adults. Infants also showed shorter median fixation durations when gazepath was used to parse the data compared to EyeLink, but the two methods produced a similar number of fixations. However, Fig. 5 also shows that there is more variance in the number of fixations classified using the gazepath method than the EyeLink method. This implies that for some infants, gazepath classified fewer fixations than EyeLink, but for others more. This is in line with the findings of the split fixations. Of the 15,368 gazepath fixations, 100 were split and 27 fixations were not classified in the EyeLink method. Of the 13,972 EyeLink fixations, 842 were split into 1005 extra fixations and 1017 were not classified in the gazepath method.

Ideally, the fixations that are split are the fixations in higher-quality data, whereas the fixations that are not classified with the gazepath method are mostly found in low-quality data. In order to see if this is indeed the case, data quality were quantified in terms of robustness and precision. Robustness was calculated as the mean length of raw data segments per trial. Infants who stay focused, have long data segments, providing a robust measure, whereas infants who look away and move a lot have many more missing data points and therefore short data segments, providing a less robust measure. To obtain the precision measure, the signal was smoothed by calculating mean x- and y-coordinates over 100-ms time windows. Precision of a trial is the mean of the mean difference between the smoothed and raw data in each time window. Low values indicate high precision and vice versa.

**Fig. 7:**
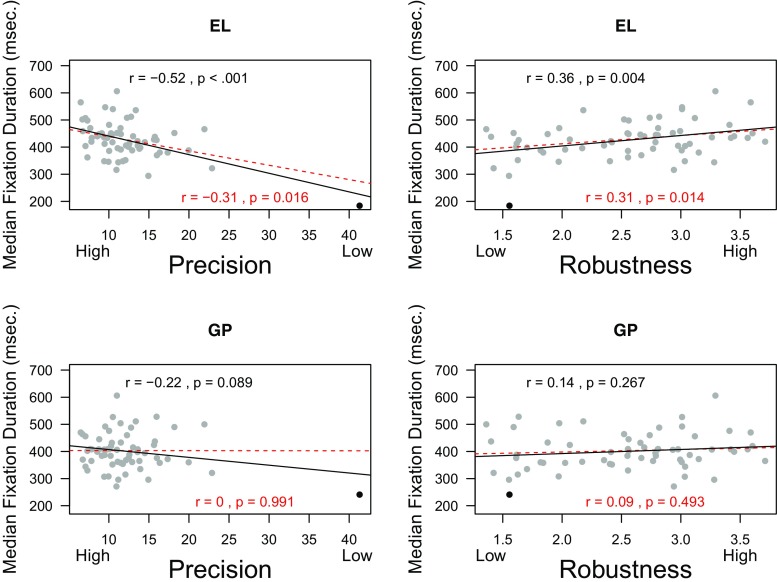
Example of data-quality measures robustness and precision and their correlations with median fixation durations classified using the EyeLink (EL) and gazepath (GP) method. Fixation durations classified by gazepath have no correlation with data quality, whereas these correlations are present with the EyeLink classification. In *red*, the correlations without the outlier are shown. The outlier is marked in *black*, which is the same data point in all plots

Correlations between data quality and fixation durations can give an indication of parsing performance. These correlations are often observed in infant data (Wass et al., 2013, 2014) and are considered problematic. As described in the introduction, these correlations can occur because poor data quality can lead to spurious short fixations. The top left panel of Fig. 7 shows the correlation (*r* = −.52, *r* = −.31 without the outlier) between precision and median fixation durations classified with EyeLink. The top right panel shows the correlation (*r* = .36, *r* = .31 without the outlier) between robustness and median fixation duration of the EyeLink classification. These correlations are significant and in the expected direction. Poor data quality is associated with shorter fixation durations when the standard EyeLink is used. The bottom left and bottom right panel show that the fixations classified with the gazepath method have correlations that are non-significant and are closer to zero. To test if these dependent correlations do indeed differ, we used a Williams test (Steiger 1980), as implemented in R-package *psych*. The Williams test showed that the correlations between median fixation duration and precision for the gazepath and EyeLink classification differed significantly with the outlier (*t*(59) = 6.52, *p* < 0.001) and without the outlier (*t*(58) = 5.84,*p* < 0.001). For robustness, similar results were obtained; the correlations between median fixation duration and robustness for the gazepath and EyeLink classification differed significantly with the outlier (*t*(59) = −3.96,*p* < 0.001) and without the outlier (*t*(58) = −3.82,*p* < 0.001). These results imply that the individual threshold estimation and post hoc checks that are implemented in the gazepath method work well.

**Fig. 8:**
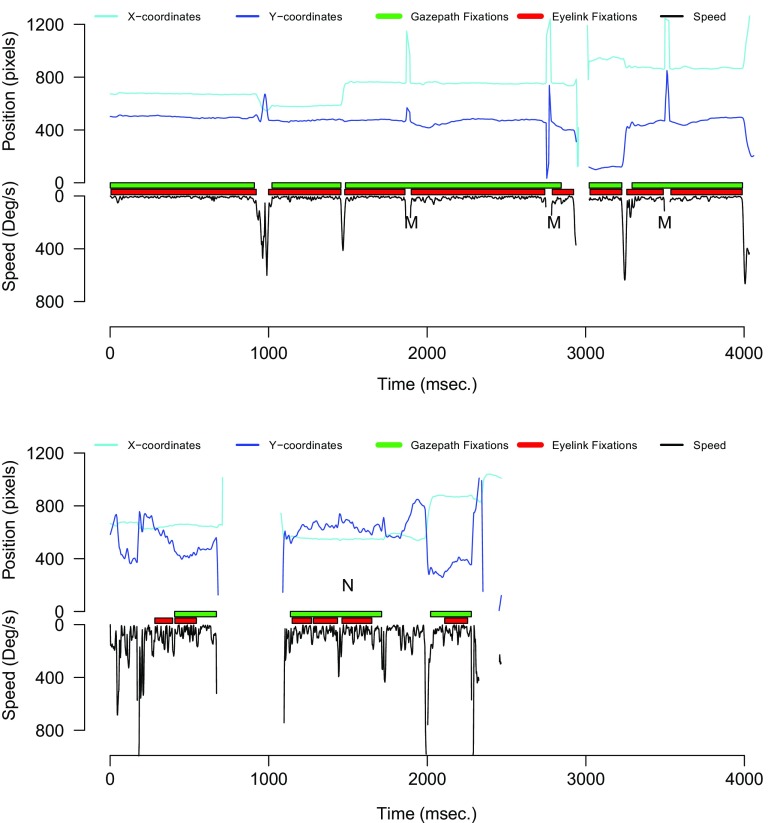
Example of gazepath and EyeLink classification for infant data. The *top panel* shows instances where data are interpolated (M). The *bottom panel* shows extremely noisy data where gazepath combines multiple EyeLink fixations into one fixation (N)

To illustrate the performance of gazepath, Fig. 8 shows two trials that are typical for what we observed in the trials with split fixations. The top panel shows instances of interpolated short missing data sequences when the X and Y position did not change (highlighted with M). The bottom panel shows a trial with very noisy data, and it can be seen that EyeLink identified multiple short fixations, whereas gazepath combined these into one larger fixation (highlighted with N). Although the bottom panel illustrates the working of gazepath, the data are extremely noisy and should probably be excluded from further data analyses.

### Conclusion free-viewing data

In this section, we showed that gazepath performs well for both infant and adult data. In high-quality adult data, gazepath lowers its thresholds and is able to pick up more fixations than the standard EyeLink method. In infant data, gazepath does the same when the infant data are of good quality, but it can also combine fixations, when low data quality or signal loss results in spuriously short fixations. Despite the good performance of gazepath, there is reason to be cautious. That is, the data sets analyzed here are the same data sets that were used to develop gazepath. It is therefore important to also examine the performance on new data sets. We selected an adult reading data set and a experimental infant data set to further examine the performance of gazepath.

## Adult reading data

To test the performance of gazepath on a data set with very different characteristics, we selected a data set of an adult reading study. A part of this data is published in experiment 2 of Koornneef, Dotlacil, van den Broek, and Sanders (2016). Reading is a highly automatic process, with predictable fixation and saccade patterns, which may make it easier to set a fixed velocity threshold. In line with what we observed in the free-viewing data, we expected gazepath to classify more and shorter fixations than the standard EyeLink method, as the individual threshold estimation allows gazepath to be more sensitive to detect short fixations.

### Participants

Sixty-five adults (*M*_*age*_ = 25.0 years, range = 18–68) participated in a reading study at Utrecht University and were paid for participating. They read 88 short texts that were 4–5 lines long. Their eye movements were measured with a EyeLink (SR Research Ltd., Ontario, Canada) eye-tracker that sampled at 500 Hz.

### Results

**Fig. 9:**
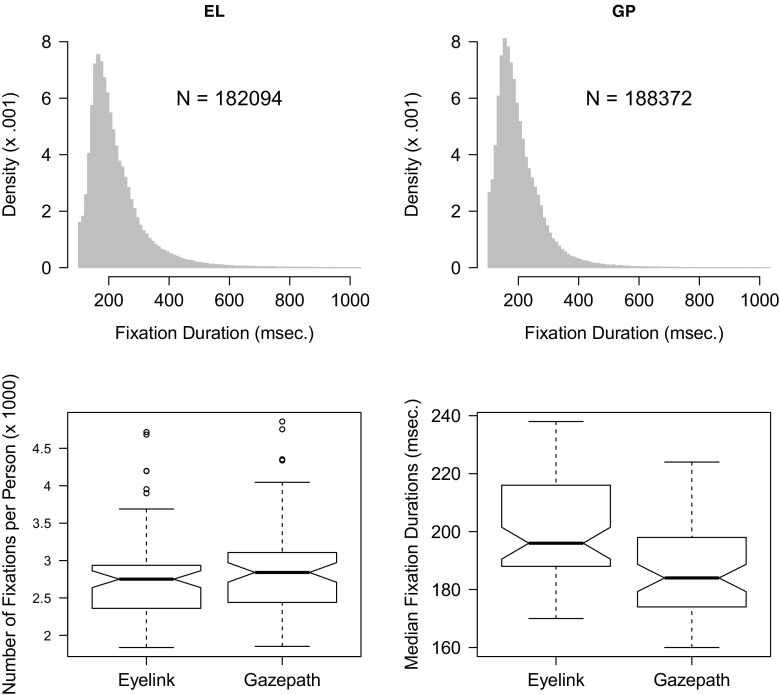
The *top panels* show the distribution of fixation durations classified with the EyeLink (EL) and gazepath (GP) method for reading data of adults. The distributions are plotted over the 100–1000-ms interval, whereas there are also some longer fixations classified. The *bottom panels* show boxplots of mean number of fixations per participant (*left*) and median fixation duration (*right*)

Figure 9 shows the distributions of the adult reading data parsed with the standard EyeLink and gazepath methods in the upper panels. The lower panels show boxplots of the mean number of fixations and median fixation durations per participant. As expected, paired-samples *t* tests showed that the gazepath method classified more (*t*(64) = −96.58,*p* < 0.001) and shorter (*t*(64) = 14.37,*p* < 0.001) fixations than the EyeLink method.

These results imply that some fixations that are classified using the EyeLink method are split into two or more fixations using the gazepath method, as was the case in the free-viewing data. To check if this is indeed what happened, we again verified for every fixation if the other method split that fixation.

Of the 188,372 gazepath fixations, only 63 were split and only 41 fixations were not classified in the EyeLink method. Of the 182,094 EyeLink fixations, 8926 were split into 9518 extra fixations and 3215 were not classified in the gazepath method. The shorter median fixation durations of the gazepath method compared to the EyeLink method can partly be explained by these splits. That is, gazepath classifies more fixations, leading to shorter fixation durations on average. However, less than 5% of the EyeLink fixations were split and therefore these splits cannot fully account for the difference. This means that there may be another difference between the two methods that also accounts for the difference in median fixation durations. For instance, there may be a difference in onset and offset times of fixations between the gazepath and EyeLink method.

**Fig. 10:**
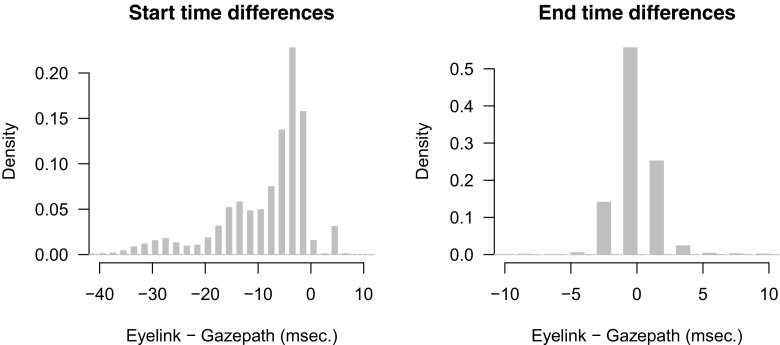
Differences (EyeLink - gazepath) between the start and end times of the same fixations, classified with EyeLink and gazepath. Both histograms are zoomed in to highlight differences around zero and show 99% of the data

To test for these differences, we selected trials (14%, *N* = 29499) that had no splits for both methods and had the exact same number of fixations. In these trials, all classified fixations are very similar and the only difference can occur in onset and offset times. In this subset of the data, we also found that gazepath had shorter median fixation durations (182) than EyeLink (194). This difference is primarily driven by later onset times of the fixations classified with gazepath compared to EyeLink. Figure 10 shows the distribution of the differences between the start (left panel) and end times (right panel) of fixations classified using the EyeLink and gazepath method. The EyeLink fixations start earlier, whereas the end times are very similar.

### Gazepath performance

**Fig. 11:**
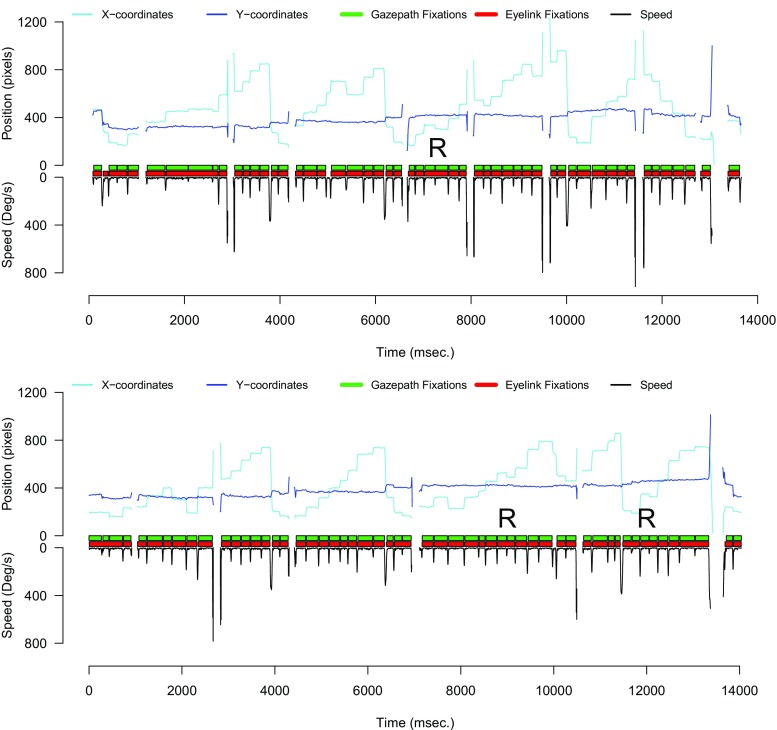
Two examples of gazepath and EyeLink classification for adult reading data. The reading pattern is clearly visible as the eye is stable on the y-axis and moves progressively higher (*to the right*) over the x-axis until the end of a sentence where a large saccade to the start of a new sentence is made. Overall, classification is very similar, although gazepath is more sensitive to detect small saccades. This can be useful for reading data as saccades in the opposite direction (R) of the reading direction (regressive saccades) are often studied

To get a better understanding of the overall performance of gazepath, we again inspected the trials that had split fixations. We observed similar patterns as in the free-viewing data of adults; gazepath is more sensitive than EyeLink to small saccades. For eye-tracking data related to reading, this can be a very useful property because saccades opposite to the reading direction are often studied. These saccades are called regressive saccades (Bicknell & Levy, 2011) and can have different interpretations. For instance, readers may miss the optimal viewing position of a word and correct with a regressive saccade (Rayner, Schotter, Masson, Potter, & Treiman, 2016). Regressions can also indicate difficulty to process a word (Vitu, McConkie, & Zola, 1998), or indicate failure to integrate a word within the context of a sentence (Frazier & Rayner, 1982). Figure 11 shows three of these instances (highlighted with R) where EyeLink missed a small regressive saccade that was picked up by gazepath.

### Conclusion reading data

EyeLink and gazepath produce very similar results when parsing adult reading data. The main difference lies in gazepath’s ability to pick up small saccades, something that can be very useful in reading studies. Another difference is that the fixations classified with gazepath are a bit shorter than fixations classified with EyeLink. This is caused by later onset times of gazepath fixations, although it is difficult to draw conclusions about one method being better than the other, as it is impossible to decide which is the ‘correct’ classification based on the eye-tracking signal alone. Overall, gazepath and EyeLink work well and produce similar results. An advantage of gazepath over EyeLink is when researchers are interested in small regressive saccades.

## Infant experimental data

To test the performance of gazepath on data of a different eye-tracker with a lower sample rate (60 Hz) and dynamic instead of static stimuli, we selected a data set of an infant experimental study using a Tobii eye-tracker. The combination of infants, a low sample rate and dynamic stimuli makes it likely that data is noisy. In line with what we observed in the infant free-viewing data, we expected gazepath to classify shorter fixations than the standard Tobii method. Given the expected noise in the data, we also expected gazepath to classify fewer fixations than the standard Tobii method, since the individual threshold estimation and post hoc checks allow gazepath to be more conservative to classify fixations in noisy data. For the same reason, we also expected to see correlations between data quality and median fixation durations classified with the Tobii method, but not with the gazepath method.

### Participants and design

The Tobii data were provided by 127 infants (*M*_*age*_ = 11 months, range = 10–12) who participated in a categorical learning study at Radboud University Nijmegen. They saw dynamic stimuli^2^ of a red ball moving to the left, or a blue ball moving to the right. The ball ended up in a cup and a reward (a small flickering chick making a whistling sound) was shown. All infants saw 20 trials of 8 s each, on a 17-inch computer monitor, which subtended an approximate 27^∘^× 34^∘^ visual angle. Eye movements were recorded with a Tobii eye-tracker (Tobii 1750, Tobii Technology, Stockholm, Sweden) that sampled at 60 Hz. Prior to data collection, a nine-point calibration scheme was used to calibrate each participant’s point of gaze.

### Results

**Fig. 12:**
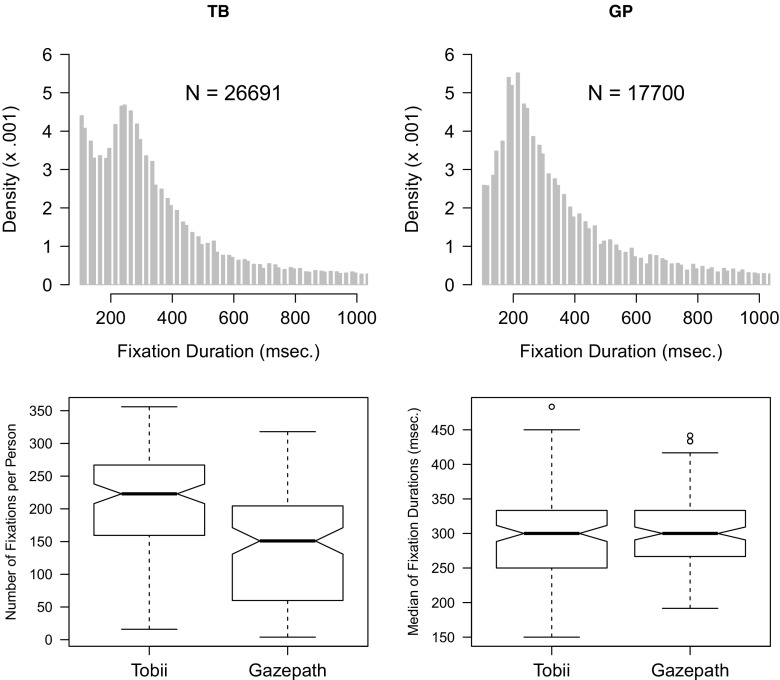
Distribution of fixation durations classified with the gazepath (GP) and Tobii (TB) method for experimental data of infants. The distributions are plotted over a 100–1000-ms interval, whereas there are also some longer fixations classified

Figure 12 shows the distributions of the infant experimental data parsed with the standard Tobii and gazepath methods in the upper panels. The lower panels show the boxplots with the mean number of fixations and median fixation durations per participant. Paired-samples *t* tests showed that the gazepath method classified fewer (*t*(126) = 13.41,*p* < 0.001), but not shorter (*t*(126) = −0.93,*p* = 0.356) fixations than the Tobii method. Of the 17,700 gazepath fixations, 902 were split into 1245 extra fixations and 133 fixations were not classified in the Tobii method. Of the 26,691 Tobii fixations, 1406 were split into 1647 extra fixations and 9600 were not classified in the gazepath method. The distribution of the Tobii fixations (Fig. 12) is oddly shaped, with many very short fixations compared to the distribution of gazepath fixations.

### Gazepath performance

**Fig. 13:**
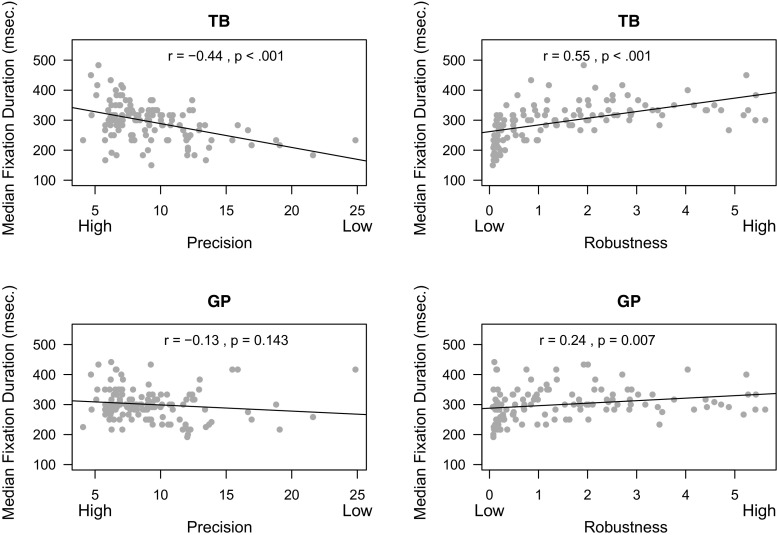
Example of data-quality measures precision and robustness and their correlations with median fixation durations classified using the gazepath (GP) method and Tobii (TB) method. Gazepath classifies more fixations in higher quality data and has lower correlations between data quality and median fixation duration than the Tobii classification

In the infant free-viewing data, we observed correlations between data quality and median fixation duration using the standard EyeLink classification method. These problematic correlations (Wass et al. 2014) were also found with the standard Tobii classification method. The upper panels of Fig. 13 show that lower precision and robustness are strongly correlated with fixation durations (*r* = −.44,*p* < 0.001 & *r* = .55,*p* < 0.001, respectively). The lower panels of Fig. 13 show that the correlation between median fixation duration and precision becomes non-significant (*r* = −.13,*p* = 0.143) and that the correlation between fixation duration and robustness becomes smaller (*r* = .24,*p* = 0.007) when gazepath is used to detect fixations. A Williams test confirmed that these correlations between median fixation duration of the gazepath and Tobii classification differed significantly for both precision (*t*(124) = 3.67,*p* < 0.001) and robustness (*t*(124) = −3.91,*p* < 0.001). This is a strong indication that the gazepath method is able to detect fixations with higher accuracy than the standard Tobii method.

**Fig. 14:**
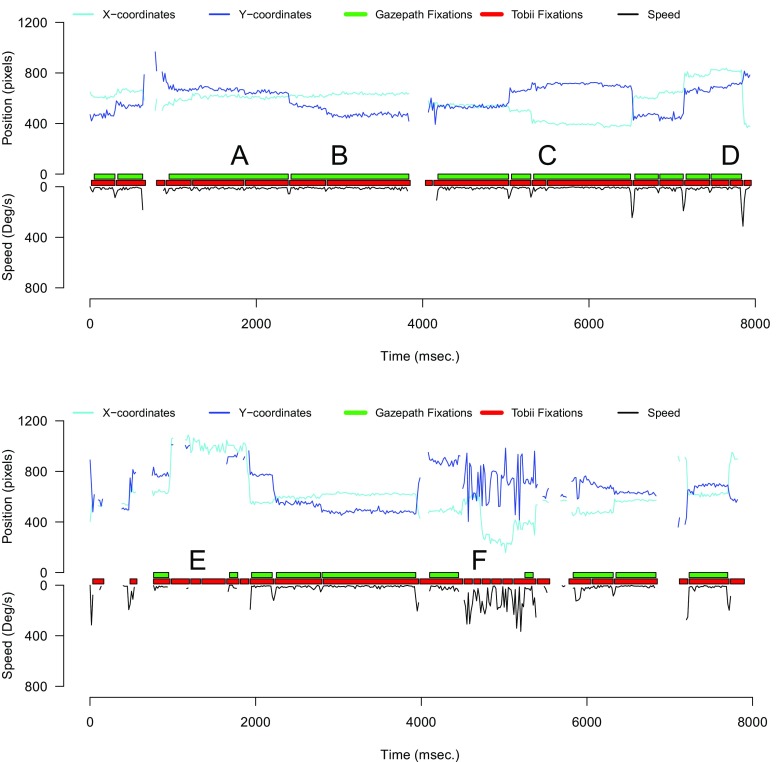
Two examples of gazepath and Tobii classification for infant data. The *upper panel* shows instances where gazepath identifies longer fixations, whereas Tobii identifies multiple short fixations (A, B, C & D). It is difficult to tell which method performed better, due to the large amount of noise. It seems that A, C, and D could be a single fixation as classified by gazepath, but they could also be multiple fixations as classified by Tobii. B is likely a double fixation that is correctly identified by Tobii, but not by gazepath. The *lower panel* shows instances (E & F) where Tobii identified fixations while there is a loss of signal from one eye (E) and extreme noise (F), and here it is clear gazepath outperforms Tobii by not classifying any fixations

To verify that the correlations between data quality and fixation durations disappeared because gazepath (1) combined fixations that should not be split and (2) correctly did not classify the 9000 fixations that were classified by Tobii, we inspected the trials that had split fixations. Figure 14 shows two trials that were typical for what we observed. There were instances where gazepath classified longer fixations, whereas Tobii classified multiple short fixations (A, B, C, & D). It is difficult to tell what classification is better, given the noise in the data. Sometimes gazepath seems too conservative; for instance B is likely two multiple fixations, instead of the one gazepath classified. The lower panel of Fig. 14 shows that gazepath does a much better job than Tobii in not classifying fixations when there is a loss of signal (E) and extreme noise in the data (F).

### Conclusion infant experimental data

In this section, we showed that gazepath also performs well in low-sampled (60 Hz), noisy infant data. The main benefit of using the gazepath method over the standard Tobii method lies in the fact that gazepath classifies far fewer fixations than Tobii. Tobii misclassified around 9000 fixations, leading to spurious correlations between fixation durations and data quality. Gazepath lowered these correlations, but could not fully account for them, as was the case in the infant free-viewing data. Finally, it seems that gazepath might still be too conservative in classifying fixations, as it remains unclear whether most long fixations classified with gazepath reflect one real underlying fixations or are actually multiple fixations.

## General conclusion

The aim of this project was to develop an easy-to-use eye-tracking data parsing tool that can be used to parse both low- and high-quality data into fixations and saccades. With the infant free-viewing data we showed how gazepath controlled for low-quality data in infants by reducing spurious correlations between fixation durations and data quality. The adult free-viewing data showed that gazepath is more sensitive than the standard EyeLink method in picking up small fixations. This finding was corroborated in the reading data set, for which we showed that gazepath can identify small fixations that are left undetected by the EyeLink method. This can be useful because small regressive saccades might be of interest in linguistic studies. Finally, we showed that gazepath also works well when parsing noisy infant data measured with a low sample rate eye-tracker and dynamic instead of static stimuli. Although gazepath seems conservative in setting its threshold, leading to (possibly too) long fixations, gazepath classified fixations better than the standard Tobii method. The largest benefit of gazepath is leaving out fixations that the Tobii method classified during loss of signal and extreme noise.

The analyses show that gazepath provides a useful tool for parsing both low- and high-quality eye-tracking data. However, it is important to note that gazepath cannot turn low-quality data into a sequence of fixations and saccades that can be interpreted perfectly. It is important that researchers inspect the data and make sensible choices about whether data can be interpreted, or data quality is too low. Gazepath’s GUI provides the user with an interface to inspect the data of all participants and trials. This makes it easy to inspect the trials with abnormally high velocity thresholds or low robustness and precision. Moreover, by providing these data-quality measures directly, gazepath makes it also easier to report such measures, something rarely seen in the literature (Hessels et al. 2015).

The gazepath method presented in this paper combines the best of several methods into one R-package. The data-driven non-parametric Mould et al. (2012) algorithm is taken as a basis to account for individual differences in data quality and looking behavior. Furthermore, modified versions of the algorithms developed by Wass et al. (2013) are used to make gazepath capable of dealing with noise typical in infant data. Finally, gazepath is implemented in R (R Core Team 2014), which is open-source software. Since gazepath comes with a Shiny app to provide a GUI, researchers can decide for themselves whether they like scripting or clicking.

## Electronic supplementary material

Below is the link to the electronic supplementary material.

(PDF 98.3 KB)

## Footnotes

1 laptop: SONY VAIO VPCEH3N1E, Intel Core i5-2450M Processor, 2.50 GHz, 4GB

2 The use of dynamic stimuli may have introduced smooth pursuit eye movements, rather than fixations and saccades only. To assess the magnitude of this possible confound, the Supplemental Materials provide the same analysis described here, without data points obtained during the dynamic part of the stimuli. In general, the analyses show similar results and overall conclusions remain the same.
