# Supplementary material for: Gazepath: An eye-tracking analysis tool that accounts for individual differences and data quality
Source: Behav Res Methods. 2017 Jun 7;50(2):834–52. doi: 10.3758/s13428-017-0909-3 (PMC5880860; doi:10.3758/s13428-017-0909-3)
Supplement: Supplementary file 1 — (PDF 98.3 KB) [file 13428_2017_909_MOESM1_ESM.pdf]

Supplemental Materials

Introduction

Apart from being collected with the Tobii and Eyelink trackers, the infant data sets also differed in the type of stimuli that were used (static vs dynamic stimuli). The use of a dynamic stimuli may explain the poor performance of the Tobii classification and may be a reason that gazepath could not fully reduce correlations between fixation durations and noise levels. Both Tobii and gazepath methods are not optimized for dynamic stimuli. To quantify the effect of dynamic versus static stimuli, we performed the same analysis as described above on a subset of the data. This was possible because the stimuli were only partly dynamic. The stimuli started static, after 1500 msec. a ball started moving, and at 4500 msec. the stimuli became static again.

Results

Figure 1 shows the distributions of the infant experimental data parsed with the standard Tobii and gazepath methods in the upper panels. The lower panels show the boxplots with the mean number of fixations and median fixation durations per participant. Paired samples t-tests showed that the gazepath method classified fewer ($t(126)$ = 14.54, $p < .001$), but not shorter ($t(126)$ = -1.25, p = 0.212) fixations than the Tobii method. Of the 10939 gazepath fixations 538 were split into 739 extra fixations and 84 fixations were not classified in the Tobii method. Of the 17188 Tobii fixations 790 were split into 909 extra fixations and 6591 were not classified in the gazepath method. The distribution of the Tobii fixations (Fig. 1) is oddly shaped, with many very short fixations compared to the distribution of gazepath fixations.

Problematic correlations between data quality and fixation durations (Wass,

Forssman, & Leppänen, 2014) were also found with the standard Tobii classification method during the time the stimuli were static. The upper panels of Figure 2 show that lower precision and robustness are strongly correlated with fixation durations (r = -.43, p < .001 & r = .55, p < .001, respectively). The lower panels of Figure 2 show that the correlation between median fixation duration and precision (r = -.17, p = .06) and between fixation duration and robustness (r = .17, p = .057) becomes non-significant when gazepath is used to detect fixations. A Williams Test confirmed that these correlations between median

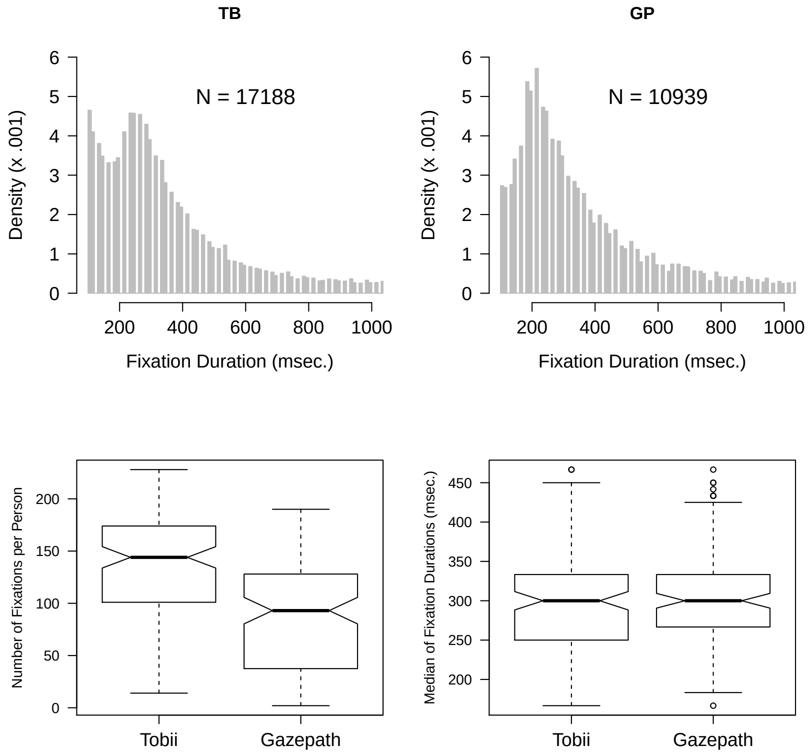

Figure 1: *Distribution of fixation durations classified with the gazepath (GP) and Tobii (TB) method for experimental data of infants. The distributions are plotted over the 100-1000 msec. interval, whereas there are also some longer fixations classified.*

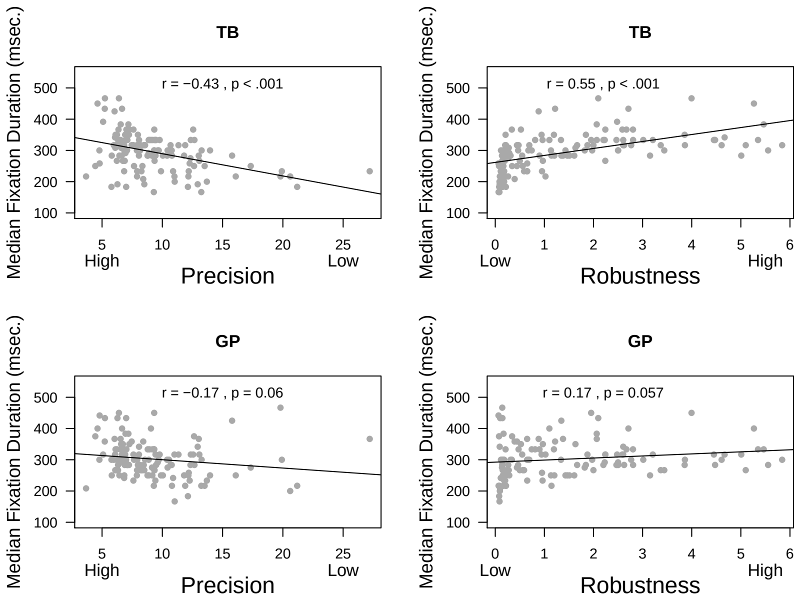

Figure 2: *Example of data quality measures precision and robustness and their correlations with median fixation durations classified using the gazepath (GP) method and Tobii (TB) method. Gazepath classifies more fixations in higher quality data and has lower correlations between data quality and median fixation duration than the Tobii classification.*

fixation duration of the gazepath and Tobii classification differed significantly for both precision ($t(124) = 2.87$, $p < .001$) and robustness ($t(124) = -4.53$, $p < .001$).

Conclusion

When only the fixations classified during the static periods are used to compare the gazepath and Tobii methods, the overall results are very similar to the results of the complete data. Although, in addition to the correlation between precision and fixation duration, the correlation between robustness and fixation duration also becomes non-significant when the gazepath method is used. For the Tobii method, on the other hand the correlations between noise levels and fixation duration remain the same.

References

Wass, S. V., Forssman, L., & Leppänen, J. (2014). Robustness and precision: How data quality may influence key dependent variables in infant eye-tracker analyses. *Infancy*, *19*(5), 427–460.
